# This Examined Life: The Upside of Self-Knowledge for Interpersonal Relationships

**DOI:** 10.1371/journal.pone.0069605

**Published:** 2013-07-31

**Authors:** Elizabeth R. Tenney, Simine Vazire, Matthias R. Mehl

**Affiliations:** 1 Haas School of Business, University of California, Berkeley, California, United States of America; 2 Department of Psychology, Washington University in St. Louis, St. Louis, Missouri, United States of America; 3 Department of Psychology, University of Arizona, Tucson, Arizona, United States of America; Vanderbilt University, United States of America

## Abstract

Although self-knowledge is an unquestioned good in many philosophical traditions, testing this assumption scientifically has posed a challenge because of the difficulty of measuring individual differences in self-knowledge. In this study, we used a novel, naturalistic, and objective criterion to determine individuals’ degree of self-knowledge. Specifically, self-knowledge was measured as the congruence between people’s beliefs about how they typically behave and their actual behavior as measured with unobtrusive audio recordings from daily life. We found that this measure of self-knowledge was positively correlated with informants’ perceptions of relationship quality. These results suggest that self-knowledge is interpersonally advantageous. Given the importance of relationships for our social species, self-knowledge could have great social value that has heretofore been overlooked.

## Introduction

What good is self-knowledge? Although self-knowledge has been an unquestioned good in many philosophical traditions, empirical research on the costs and benefits of self-knowledge paints a more complicated picture. Much of this research has focused on the intrapsychic consequences of self-knowledge (e.g., for happiness or mental health) and found mixed results [1] [2] [3] [4] [5]. However, another place to search for the benefits of self-knowledge is in the interpersonal domain. How does having self-knowledge affect what other people think of you? We first define self-knowledge and then discuss evidence of its interpersonal costs and benefits.

### What is Self-Knowledge?

Self-knowledge is defined as accurate self-beliefs [6]. Accuracy has been a notoriously thorny phenomenon to measure in psychological research [7], but conceptually it refers to the correspondence between a judgment (in this case a self-judgment) and reality. Thus, self-knowledge is the degree to which a person’s self-views correspond to what she or he is actually like. People can have self-knowledge about momentary states or stable dispositions across a variety of constructs including emotions, attitudes, behaviors, traits, goals, motives, and autobiographical memories (see, e.g., [8]). In this paper we focus on the accuracy of self-views about stable patterns of behavior.

Another important definitional issue in the study of self-knowledge is defining its opposite. It may seem obvious that if self-knowledge is defined as accurate self-beliefs, its opposite is inaccurate self-beliefs. However, matters get complicated when thinking about all the different ways self-beliefs can be inaccurate. As the large literature on bias in social psychology shows [9], there are many ways to be inaccurate. For example, the literature on self-enhancement shows that people can have overly positive self-views. Indeed, much of the literature on self-knowledge focuses specifically on self-knowledge compared to positive self-biases (e.g., overconfidence, self-enhancement).

What can the literature on positive self-biases tell us about self-knowledge? In some ways, a great deal. If we interpret the absence of positive self-biases to be an indication of self-knowledge, then the consequences of self-enhancement can be interpreted as the consequences of self-knowledge (with the direction of the associations reversed). Indeed, many researchers interpret their findings this way (e.g., [10]). On the other hand, there is the worry that people scoring low on individual difference measures of self-enhancement may be just as self-deluded as those scoring high, but in the opposite direction. They may have self-views that are more negative than reality. In that case, the consequences of self-enhancement cannot be assumed to apply (in reverse) to self-knowledge [11]. There is little evidence about whether people scoring low in self-enhancement are accurate or are self-diminishing. Thus, the literature on positive self-biases must be interpreted with caution when drawing conclusions about self-knowledge. Nevertheless, it is the largest body of research relevant to the costs and benefits of self-knowledge, so we now review what conclusions can, cautiously, be drawn from this literature.

### The Costs and Benefits of Self-Knowledge

There is reason to believe that self-enhancement may be associated with better interpersonal outcomes than self-knowledge. Specifically, several researchers have argued that self-deception helps people deceive others. The claim is that if a person truly believes that she is better than she really is, then when she presents herself to others, she will act in ways (e.g., confident about her abilities) that make others likely to believe she is better than she really is, too [12] [13] [14]. Thus, people with overly positive self-views will make more positive impressions on others than will people with accurate self-views. In line with this hypothesis, one recent set of studies found that overconfident people were perceived by others as more capable and higher status than people with accurate self-views [15]. In these studies, perceivers likely could not tell when a person was justified or unjustified in holding positive self-views because people with unjustified positive self-views exhibited behavioral cues that made them seem highly competent (e.g., a calm and relaxed demeanor, a confident and factual tone of voice). They did not make bold claims (e.g., “I am good at this task!”) that could easily be refuted. Thus, self-enhancement may have positive interpersonal consequences because others are likely to buy into a person’s self-delusions.

Many of the findings supporting the social benefits of self-enhancement, however, suffer from a methodological difficulty. Specifically, the operationalization of interpersonal outcomes (such as relationship quality) affects conclusions about whether self-enhancement is advantageous. While positive interpersonal consequences of self-enhancement were found when interpersonal outcomes were measured with self-ratings, these findings have been found not to replicate (and to reverse) when interpersonal outcomes were measured with peer- or observer-reports (e.g., [16] [3]). The reason for the different findings using self- vs. other-reports could be that self-reports of self-enhancement share method variance with self-reports of interpersonal adjustment, and without proper controls, the correlations observed are therefore inflated [4] [17] [18]. People who report being better than they really are in terms of their abilities or personality might also report having better relationships with others than they really do.

Indeed, there are a number of studies suggesting that self-enhancement carries important interpersonal costs. Although overconfidence is beneficial when indistinguishable from confidence, overconfidence can be damaging to people’s reputations if discovered [19] [20] [21]. In a set of studies, confident job applicants vying for a position initially fared better than less confident job applicants. However, the confident applicants lost ground in the application process after it was revealed that they had been too confident about their abilities. In contrast, applicants did not lose ground if they had exhibited self-knowledge, rather than excessive confidence, about their strengths and weaknesses [20].

Studies also show that self-enhancement can lead to deteriorating interpersonal relationships over time. For example, self-enhancers make a positive first impression, but they come to be seen negatively after repeated interactions [22] [23]. Self-enhancers also tend to have poor social skills [24] and are disliked by others [25] [26]. Furthermore, people who overestimate their status in a group are disliked by their group members [27] [28]. These results suggest that self-deception may be detrimental to maintaining good relationships.

### What’s so good about Self-Knowledge?

One aim of this paper is to provide a rigorous test of the relationship between self-knowledge and the quality of close relationships. Based on the literature reviewed above, we predict that self-knowledge will be associated with good relationships. A secondary aim of this paper is to shed light on potential explanations for this predicted association. Given that we know relatively little about the interpersonal consequences of self-knowledge, discussion of the mechanisms underlying these consequences is necessarily speculative at this point. Nevertheless, we propose one potential explanation: that self-knowledge is valuable for its own sake in close relationships.

Certainly, self-knowledge is likely to have instrumental value–there are probably some practical benefits of having self-knowledge. For example, self-knowledge is likely associated with making better (or better-fitting) life decisions that likely make a person more pleasant to be around [29] [30]. However, we propose that self-knowledge also has intrinsic value, an idea that goes back at least to Aristotle, who postulated that self-knowledge of one’s personality or character is a virtue in itself, apart from its ability to lead to virtuous actions [31]. That is, independent of the practical benefits of self-knowledge, people value self-knowledge in themselves and others. Specifically, we predict that the link between self-knowledge and good relationships is direct and not fully explained by the practical benefits of self-knowledge.

We test this hypothesis by demonstrating that the relationship between self-knowledge and relationship quality is independent of other potential benefits of self-knowledge. If self-knowledge is valued for its own sake, self-knowledge should predict relationship quality, and this effect should persist after controlling for positive personality traits (e.g., agreeableness, emotional stability). These findings would show that the relationship between self-knowledge and better relationships is not due to people with greater self-knowledge having more positive qualities.

### The Current Study

The purpose of the current study was to examine whether self-knowledge is associated with better interpersonal relationships and whether this effect holds when controlling for positive qualities that may be confounded with self-knowledge. We examined the interpersonal consequences of self-knowledge using a novel, objective measure of self-knowledge and a peer-report-based measure of interpersonal adjustment. Nearly all studies examining individual differences in self-knowledge face tricky definitional issues with the operationalization of self-knowledge. Both of the two most commonly used methods for calculating individual differences in self-enhancement have important confounds (as noted by [32] [4] [24]). The social comparison approach (asking participants to rate themselves relative to others) confounds self-enhancement with actually possessing positive traits, because people who rate themselves more positively than they rate others may not be self-enhancers if they in fact have exceptional personalities. Conversely, the self-insight approach (comparing self-reports to observer-reports of the target) confounds self-enhancement with the tendency to see everyone (including the self) positively, because people who rate themselves more positively than others rate them may not be self-enhancers if they simply have an especially rosy view of all people (including themselves).

Kwan et al. (2004) proposed an alternative measure of self-enhancement based on the social relations model (SRM) to get around these confounds [4]. Although their proposed SRM-based measure of self-enhancement is a great improvement over the two traditional approaches and is ideal for many purposes, it is potentially ill-suited for examining the interpersonal consequences of self-knowledge. While the SRM-based measure is ideally suited for measuring discrepancies between self-views and others’ views of a person, we were interested in measuring the accuracy of self-views against a criterion that did not include others’ perceptions. Because the goal of the current study is to examine whether self-knowledge is associated with better interpersonal relationships, it is important that the measure of self-knowledge not be based on the interpersonal perceptions from the same people who are also rating the quality of the relationship. If both the self-knowledge measure and the relationship quality measure involve ratings by the same peers, the shared method variance could inflate the correlation between the two variables. Thus, to examine the interpersonal consequences of self-knowledge, a different approach is needed.

To do so, we chose to assess the accuracy of people’s self-views about their typical daily behavior. Knowledge of how one behaves in everyday life is, admittedly, only one form of self-knowledge. However, daily behaviors are the ingredients of personality [33] and, as such, knowing one’s daily behavior is an important component of self-knowledge [34] [35]. For example, a person who believes that she is exceptionally sociable (spends a lot of time with others, talks a lot, laughs a lot), but actually spends much more time alone and silent than other people, could be exhibiting important blind spots in her self-knowledge.

In order to obtain a criterion measure that was both objective (i.e., independent from self-views, but also independent from peer-reports), and ecologically valid (i.e., reflects what people are actually like in their everyday lives; see [36], and [37], on the benefits of measuring behavior in natural contexts), we collected audio recordings of people’s actual daily behavior using the Electronically Activated Recorder (EAR; [38]). Thus, we operationalized self-knowledge by comparing people’s beliefs about how they typically behave to their actual daily behaviors observed in natural contexts via unobtrusive sampling of audio recordings. We then used an idiographic, profile correlation approach to compute individual-level self-knowledge scores. Our design overcomes many of the obstacles that have hampered past efforts to study self-knowledge empirically by 1) using an objective, ecologically valid criterion against which to compare self-views, 2) avoiding the “social comparison” and “self-insight” indices of self-knowledge that have important confounds, and 3) using an outcome measure (informants’ ratings of relationship quality) that does not share method variance with the self-knowledge measure.

In this study, we had three main objectives. First, we aimed to introduce a methodology for measuring self-knowledge using an objective, ecologically valid criterion. Second, we aimed to show that, compared to people with poor self-knowledge, people with greater self-knowledge have close others who report having better relationships with them. Finally, we aimed to provide preliminary evidence that the association between self-knowledge and better relationships stems from the fact that people value self-knowledge for its own sake, not because of desirable personality traits that might be associated with self-knowledge. We expected that, if self-knowledge has intrinsic value, then self-knowledge would be positively correlated with relationship quality and that personality desirability would not mediate this link.

## Method

### Participants

Eighty undergraduates (43 women, 37 men; *Mdn*_age_ = 18) recruited mainly from Introductory Psychology courses completed self-ratings and nominated three informants: one parent, their closest friend, and one romantic partner if possible. One-hundred-eighty-two informants responded (106 women, 73 men, 3 no gender reported). Participants received $50 for participating. Informants were not compensated. Three participants had to be excluded from analyses due to missing data. The study was approved by the University of Texas, Austin Institutional Review Board. Participants provided written informed consent. This study was part of a larger study on self and other perceptions of personality and behavior. Portions of these data were published in [39] [40] [41] [42]. The analyses of the current research questions do not overlap with any of those published elsewhere.

### Materials and Procedure

During a lab session, participants nominated three informants, and informants were contacted via email. Participants were told that they would never see the ratings that informants made about them. Participants and informants rated participants’ behavior on the ACT questionnaire [42]. The ACT assesses self- and other-perceptions of the frequency of daily behaviors (e.g., laughing, talking, typing, commuting, watching TV). All items on the ACT refer to overt, observable behavior (See Appendix S1 for a list of the items used in our analyses). On the participant-version of the ACT, participants were asked, “Compared to other people, how much do you do the following activities?” whereas on the informant-version of the ACT, informants were asked “Compared to other people, how much does X do the following activities?” with X representing the target participant. Aside from this wording in the instructions and changing the second-person pronoun to the third-person (e.g., “you” became “he/she”) in the item wording, the participant and informant versions of the ACT were identical. Ratings of how much participants engaged in the daily activities specified on the ACT were made on Likert-type scales from (1) *much* less *than the average person* to (7) *much* more *than the average person.* It is worth noting that, by design, these items asked participants and informants to make comparative judgments, wherein accuracy depended on both accurate assessments of a participant’s behavior and participant’s standing relative to others (see [43] and [44] for related discussions). The comparative approach is appropriate here because personality is inherently relative–to be a warm, friendly person means to be more warm and friendly than the average person. Knowing the absolute frequency of behavior (e.g., “I spend 9.4 hours per day with others”) without accurate awareness of where this puts someone relative to others (e.g., “I am more sociable than the average person”) is arguably a less useful or relevant type of self-knowledge (see [15] and [45] for examples of others using a comparative approach to study self-knowledge). We also could have used non-comparative Likert scales asking people how often they engaged in certain behaviors from, for example, *never* to *very often,* or how much they believed certain traits characterized their behavior from *not at all* to *definitely*. But then it would have been virtually impossible to determine objectively whether participants were accurate in their self-assessments or not. There would be no clear appropriate benchmark to which their judgments could be compared.

Participants then wore an Electronically Activated Recorder (EAR; [38]) for four days, which was a small digital audio recorder that automatically and periodically (every 12.5 min) sampled 30 seconds of ambient sounds around participants. Participants had no way of telling when the device was and was not recording. Compliance with instructions to wear the EAR during all waking hours save when the device could be harmed (e.g., when showering) was high. Participants reported having it on them for an average of 72% (*SD* = 16%) of their time awake, yielding an average of 300 (*SD* = 104) valid audio recordings per participant. Participants were given the opportunity to listen to their sound files and delete any of the recordings before turning them over to the experimenter. Only very few recordings were deleted (<0.01%). A team of ten coders listened to participants’ EAR recordings and coded them for acoustically detectible signs of each ACT item (for detailed coding instructions, see [38] and [46]). Coders used acoustic cues such as the noise of a running engine, the voice of someone lecturing, or sounds of typing to code participants’ activities. In addition, coders used contextual information from previous and consecutive intervals to increase their accuracy (e.g., the noise of a large engine followed by someone lecturing would indicate that a student rode a bus to get to class). Each participant’s sound files were coded by one coder. As in previous studies (e.g., [42]), we included only the 17 items on the ACT that could be detected and coded reliably using the EAR. Intercoder reliabilities were determined from a set of training EAR recordings (221 sound files) that was independently coded by all 10 research assistants. ICC[2,k] exceeded.70 for all categories (See Appendix S1 for a list of the items). For each participant, the binary EAR codings (i.e., behavior present or absent) were aggregated across all sound files into a relative frequency measure (i.e., the percentage of sound files in which a behavior was displayed; see [38] for more information on coding procedures). We then converted these relative behavior frequencies into z-scores across the sample (individually for each behavior). These data, coded from the EAR sound files, formed the criterion measure of how participants actually behaved in daily life.

Informants, in addition to rating participants’ behavior on the ACT questionnaire, also gave their impression of participants’ personality, attractiveness, and intelligence, and they rated their relationship with the participant. Specifically, informants assessed participants’ personality using the Big Five Inventory [47] and rated participants’ attractiveness and intelligence on scales from 1 to 7. The measure of relationship quality asked informants to rate closeness, relationship quality, and liking on scales from 1 to 7. As might be expected, most informants reported having good relationships with participants, creating a distribution with a negative skew (–2.1). Transformation to the fourth power successfully reduced the skew to within two standard errors of skewness (–.42) and did not meaningfully change any effects reported here.

Like informants, participants also rated relationship quality. They rated “How close are you and [the informant]” and “How would you rate the quality of your relationship with [the informant]?” but unlike the informants, participants did not rate liking. We report the main analyses both with (α = .82) and without (α = .88) the participants’ ratings included in the relationship quality index. The indices yield the same conclusions, but we made the informant-only index the primary dependent variable because it does not share any method variance with the independent variable (self-knowledge).

## Results and Discussion

### Does Self-Knowledge Predict Relationship Quality?

**Figure 1:**
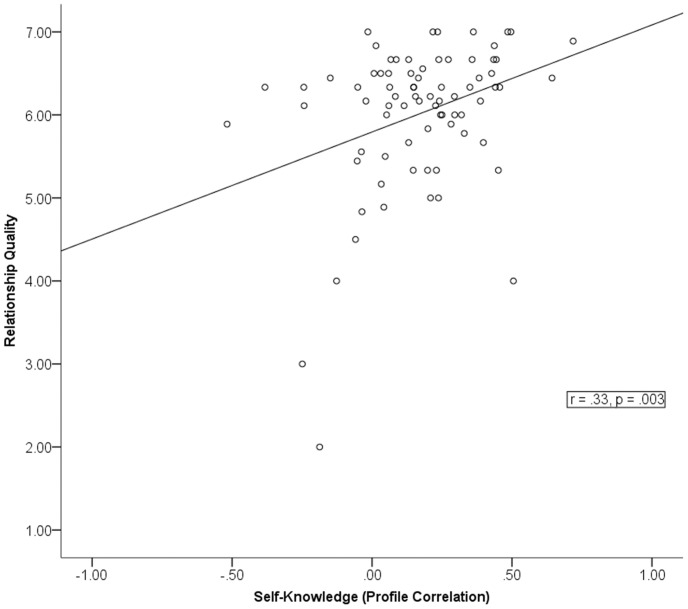
Relationship quality as a function of participants’ degree of self-knowledge (N = 77). Figure 1 shows the association between individual differences in self-knowledge and informant-rated relationship quality. Self-knowledge was operationalized as how well participants knew how they typically behaved in daily life compared to others. In order to compute a self-knowledge score for each participant, we calculated each participant’s profile correlation between self-ratings of daily behavior (on the ACT questionnaire) and behavioral codings of actual daily behavior (from the EAR sound recordings) across 17 items. Relationship quality was the mean of informants’ ratings of relationship quality, closeness, and liking.

Our aim was to test whether self-knowledge of daily behavior was associated with good relationships. We predicted that, compared to people with poor self-knowledge, people with greater self-knowledge would have close others who report having better relationships with them. Self-knowledge was operationalized as how well participants knew the relative frequency of various behaviors they performed in daily life. In order to compute a self-knowledge score for each participant, we calculated each participant’s profile correlation between self-ratings of daily behavior (on the ACT) and behavioral codings of actual daily behavior (from the EAR sound recordings) across 17 items. Profile correlations are straightforward ways of quantifying agreement between sets of items or behaviors by comparing the pattern of highs and lows in each set of items; that is, the shape of the profiles [44]. We computed a self-knowledge profile correlation for each participant and these correlations, converted to Fisher z values, ranged from –.52 to.72 (*M* = .17, *SD* = .22). (We did not compute distinctive profile correlations [44] because one variable (actual behavior) was standardized, so the profile correlations could not be driven by agreement with a “typical” or normative profile (cf. [48])). Higher profile correlations indicate greater self-knowledge. For example, if behaviors participants viewed as relatively descriptive of how they spent their time (as compared to other behaviors) were the same behaviors that the EAR recordings also revealed were relatively descriptive of how they spent their time, participants would have high self-knowledge. Individual differences in self-knowledge were positively associated with informant-rated relationship quality (*r* = .33, *p* = .003; Figure 1), showing that more self-knowledge was in fact associated with better relationships.

We also explored whether self-knowledge was correlated with each of the three items in the relationship quality index separately (the items measured closeness, liking, and relationship quality, α = .88). Self-knowledge was significantly positively correlated with each item, all *r*s >.28, all *p*s ≤.013. To explore the potential effect of outliers, we excluded participants who scored at least 1.5 times the interquartile range on either self-knowledge or relationship quality (*n* = 7). The size and significance of the relation between self-knowledge and relationship quality did not meaningfully change (*r* = .25, *p* = .039) suggesting that outliers exerted little impact on the effect.

Because relationships are dyadic, we also created a version of the relationship quality index that, in addition to informants’ ratings, included participants’ ratings of closeness and quality of relationship. This index shared some method variance with self-knowledge because the same participants rated both their self-perceptions of their behavior (one half of the self-knowledge profile correlation) and relationship quality. This index was positively correlated with self-knowledge (*r* = .23, *p* = .048) and corroborated the effects found with the informant-only measure of relationship quality.

### Potential Indirect Effects

We tested whether people with greater self-knowledge had better relationships independent of the desirability of their personalities. Recall that we predicted that self-knowledge has intrinsic value, and thus self-knowledge should remain a significant predictor of relationship quality even after controlling for other positive characteristics. Consistent with this hypothesis, individual differences in self-knowledge were not significantly correlated with informant-ratings of personality, attractiveness, or intelligence (see Table 1). Furthermore, the relationship between self-knowledge and relationship quality remained significant and positive after controlling for these variables in a single multiple regression (*β* = .206, *p* = .001) suggesting that the relationship between self-knowledge and relationship quality is robust and independent of other characteristics. These results are consistent with the hypothesis that self-knowledge has value on its own.

**Table 1:** Means, Standard Deviations (SD), and Correlations (r) with Self-knowledge and Relationship Quality.

Variable	*Mean (SD)*	*r* with Self-Knowledge	*r* with Relationship Quality
Extraversion	4.8 (1.1)	–.07	.28*
Agreeableness	5.1 (1.1)	.21	.63**
Conscientiousness	4.9 (.95)	.07	.31**
Emotional Stability	4.3 (1.0)	.16	.48**
Openness	5.0 (.92)	.04	.33**
Attractiveness	5.5 (1.4)	.10	.75**
Intelligence	6.4 (0.7)	.06	.38**
Informant-Knowledge	.18 (.19)	.27*	–.12
Relationship Quality	6.0 (.88)	.33**	–

Note: **p<.01; *p<.05. N = 77. All variables are informant-reported on 7-point Likert-type scales except self-knowledge and informant-knowledge. Self-knowledge is the profile correlation between self-reported behavior and actual behavior. Informant-knowledge is the profile correlation between informant-reported behavior and actual behavior.

We next tested the possibility that people with greater self-knowledge were more predictable, and that predictability, rather than self-knowledge, accounted for their better relationships. Perhaps the typical behavior of people who had greater self-knowledge was actually easier for anyone to know, not just the self. In other words, we wanted to explore if people liked others with self-knowledge simply because people with self-knowledge acted in typical or predictable ways. We were also concerned that what looked like self-knowledge could simply be an artifact of being predictable (i.e., anyone could predict this person’s behavior) rather than the result of insight unique to the self. Therefore, it was important to examine potential effects of predictability (or “knowability”) on relationship quality. If our effect is actually due to predictable people having better relationships, then informants who had more accurate perceptions of participants’ daily behavior on the ACT (e.g., time spent talking, laughing, or attending class) would report higher relationship quality with participants. To test the predictability explanation, we computed an informant-knowledge score that was exactly analogous to the self-knowledge score for each participant. In other words, the informant-knowledge score was computed as the profile correlation between informant-ratings of behavior (on the ACT) and behavioral codings of actual daily behavior (from the EAR recordings) across 17 items, then converted to Fisher z values. This informant-knowledge score represents how predictable participants were to the informants. We examined whether informant knowledge was associated with relationship quality. The fact that it was not (*r* = –.12, *p* = .283) provides evidence against the explanation that the results were driven by predictability rather than self-knowledge. Furthermore, self-knowledge remained a significant predictor of relationship quality when controlling for informant-knowledge in a regression (*β* = .391, *p* = .001), which suggests that predictability did not drive the effect of self-knowledge on relationship quality.

Another consideration is whether participants knowingly changed their behavior to be consistent with their ACT answers, which would suggest that our measure of self-knowledge actually measured individual differences in the extent to which participants were intentionally acting in line with their self-views while wearing the EAR. This prospect is unlikely for several reasons. First, participants did not know that their answers would be compared to their actual behavior recorded with the EAR, mitigating the demand to appear consistent. Second, participants completed other questionnaires unrelated to the current analyses during the session in which they completed the ACT, making it unlikely that they could monitor their behavior to act in accord with the answers they provided on all questionnaires. Third, some items were not entirely under participants’ control (e.g., time spent commuting). Fourth, because participants did not know when the recorder was on, they would have had to monitor their behavior constantly for four days. Therefore, we are confident that participants who accurately reported how they typically behave possessed self-knowledge rather than a proclivity to change their behaviors to fit their answers.

Thus, our findings show that self-knowledge of behavior is associated with better relationships. Furthermore, by ruling out several potential alternative explanations for this association, we have presented evidence supporting the notion that self-knowledge may have an independent, direct effect on relationship quality. That is, self-knowledge may have intrinsic value for interpersonal relationships.

## General Discussion

This study showed that people have good relationships with others who possess self-knowledge of their daily behavior. This effect existed even when controlling for the fundamental tendency to rate people with desirable personality traits as good relationship partners [49]. We measured self-knowledge by comparing people’s self-reports to an objective, ecologically valid criterion. Perceivers reported having better relationships with people who possessed self-knowledge, measured objectively, than with people who lacked self-knowledge. Thus, knowing oneself is a marker of good relationships.

### Potential Mechanisms

Why *do* self-knowledgeable people have better relationships? One possibility that we sought to provide support for is that self-knowledge has intrinsic value–or, in Aristotle’s words, is a virtue–and people prefer others who possess this virtue. In other words, we proposed that there is a direct relationship between self-knowledge and liking because self-knowledge is itself a desirable quality. The results are consistent with this explanation. When someone had self-knowledge, informants reported having better relationships with her or him. This effect held even after controlling for other positive characteristics like informants’ ratings of intelligence, emotional stability, and agreeableness. Self-knowledge continued to predict relationship quality after controlling for perceptions of people’s other positive qualities.

Of course, ruling out one potential set of instrumental benefits does not prove that self-knowledge has intrinsic value. There are other potential practical benefits of self-knowledge that may account for its link to relationship quality. For example, self-knowledge may confer a diffuse set of skills, not captured by broad traits, that are conducive to good relationships. For example, people with self-knowledge might be in a better position to make appropriate decisions for themselves or recognize and utilize their strengths and weaknesses. Our findings show that the link between self-knowledge and relationship quality cannot be accounted for by Big Five traits, attractiveness, or intelligence (informant-rated), but perhaps these constructs do not capture some of the practical advantages of self-knowledge. Discovering precisely which aspects of personality or behavior self-knowledge bolsters or enables to create better relationships, if any, is worthy of further empirical inquiries.

Finally, the causal arrow may go in the other direction–people with strong relationships may have greater self-knowledge because their strong relationships help them learn about themselves (e.g., through direct or indirect feedback; [50] [51] [52]). We suspect that all three of these processes (intrinsic value, instrumental value, and reverse causation) play some role in explaining why self-ignorant people are less well-liked–self-knowledge likely has both intrinsic and instrumental value, and it may lead to a “virtuous cycle” whereby good relationships help to foster even better self-knowledge.

### Conclusions and Future Directions

Is self-knowledge good? Although this study cannot speak to its intrapsychic costs or benefits, our results show that self-knowledge seems to be related to important interpersonal benefits. This study cannot and should not end the debate about the benefits of self-knowledge versus various types of self-deception, but the findings presented here raise several important and new possibilities. First, as researchers and theorists are beginning to demonstrate (e.g., [15] [20] [14]), the consequences of self-knowledge and self-deception are likely not limited to the self. People’s traits and behaviors form the foundation of their interpersonal interactions and relationships, and the consequences of people’s awareness (or lack thereof) of these traits and behaviors also likely extend to their social worlds. Thus, although the study of self-knowledge is inherently self-focused, it is likely a fruitful avenue for examining the antecedents of interpersonal outcomes such as relationship stability and satisfaction, social network dynamics, and occupational outcomes. We hope future research will examine whether self-knowledge is associated with positive or negative outcomes across a wide range of interpersonal domains.

Second, as is often the case, obtaining ecological validity comes at the price of increased effort for participants, coders, and experimenters, but for certain questions, is a worthwhile endeavor [36]. Given the challenges inherent to measuring self-knowledge (e.g., finding an accurate, appropriate benchmark against which self-views can be compared), the approach used here could serve as a blueprint for other operationalizations of self-knowledge. Specifically, profile correlations between a set of self-views and a set of objective, naturalistic criterion measures are a promising way to measure individual differences in self-knowledge while avoiding many of the confounds inherent to the social comparison and self-insight approaches. The EAR is a powerful tool for obtaining objective and naturalistic criterion measures and can be used to study a diverse range of behaviors and populations. Future research should examine the convergence between this and other operationalizations of self-knowledge (e.g., Kwan et al.’s SRM-based approach [4]).

The results presented here also point to the need for more comprehensive examinations of self-knowledge and interpersonal processes. For example, a longitudinal approach would shed light on the development of self-knowledge over time and the causal relationships between self-knowledge and interpersonal adjustment. Does self-knowledge increase throughout adulthood? Do increases in self-knowledge lead to improvements in social connectedness? What kinds of self-knowledge are important and across what domains? We do not know if self-knowledge functions similarly in all situations.

Finally, the results presented here also highlight the need for more controlled investigations into the causes and consequences of self-knowledge. What interventions or experimental manipulations might increase self-knowledge and affect subsequent social interactions? Inquiries about the consequences of self-knowledge can be traced back to Socrates, Freud, and other classic philosophical thinkers, but a unified investigation of self-knowledge in social and personality psychology is new, and many questions remain [8] [35]. Given the extraordinary importance of interpersonal relationships for our social species [53] [54], self-knowledge could have great social value that has heretofore been overlooked.

## Supporting Information

Appendix S1 **ACT items in analyses.**
(DOCX)
