# Supplementary material for: This Examined Life: The Upside of Self-Knowledge for Interpersonal Relationships
Source: PLoS One. 2013 Jul 31;8(7):e69605. doi: 10.1371/journal.pone.0069605 (PMC3729952; doi:10.1371/journal.pone.0069605)
Supplement: Appendix S1 — ACT items in analyses. (DOCX) [file pone.0069605.s001.docx]

**Appendix**

ACT items in analyses

| 1. Spend time with other people |
| --- |
| 1. On the phone |
| 1. Talking one-on-one |
| 1. Talking in a group |
| 1. Talking to the same sex |
| 1. Talking to opposite sex |
| 1. Laughing |
| 1. Singing |
| 1. Listening to music |
| 1. Watching TV |
| 1. On the computer |
| 1. Attending class |
| 1. Socializing |
| 1. Indoors |
| 1. Outdoors |
| 1. Commuting |
| 1. At coffee shop/bar/restaurant |
